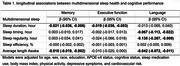# Multidimensional sleep health and longitudinal cognitive performance

**DOI:** 10.1002/alz70857_107258

**Published:** 2025-12-25

**Authors:** Hui Shi, Yunyi Sun, Derek B. Archer, Arden Perry, Kimberly R. Pechman, Dandan Liu, Timothy J. Hohman, Angela L. Jefferson, Kelsie M Full

**Affiliations:** ^1^ Vanderbilt Memory and Alzheimer's Center, Nashville, TN, USA; ^2^ Vanderbilt University Medical Center, Nashville, TN, USA; ^3^ Vanderbilt Genetics Institute, Vanderbilt University Medical Center, Nashville, TN, USA; ^4^ Department of Neurology, Vanderbilt University Medical Center, Nashville, TN, USA; ^5^ Vanderbilt Brain Institute, Vanderbilt University Medical Center, Nashville, TN, USA; ^6^ Vanderbilt Memory and Alzheimer's Center, Vanderbilt University Medical Center, Nashville, TN, USA; ^7^ Vanderbilt Memory and Alzheimer's Center, Vanderbilt University Medical Center, Nashville, TN, USA; ^8^ Vanderbilt Memory and Alzheimer's Center, Vanderbilt University School of Medicine, Nashville, TN, USA; ^9^ Department of Biostatistics, Vanderbilt University Medical Center, Nashville, TN, USA; ^10^ Department of Medicine, Vanderbilt University Medical Center, Nashville, TN, USA

## Abstract

**Background:**

Poor sleep health has emerged as a potentially modifiable risk factor for Alzheimer's disease (AD) and related dementias (ADRD). Previous studies have mainly focused on self‐reported sleep duration or a single measure of sleep health with cognitive performance. In a cohort of older adults with comprehensive neuropsychological assessment, we investigated actigraphy‐measured sleep health with cognitive trajectories over a 9‐year period.

**Method:**

Participants from the Vanderbilt Memory and Aging Project (*n* = 553, Mean age: 68.5± 9.3 year; 50.3% women) wore ActiGraph GT9X accelerometers on their wrist daily for 10 days. Data were processed for estimates of sleep duration, timing, regularity, efficiency and several measures of fragmentation. We used linear mixed effects models to assess the associations between baseline sleep measures and longitudinal changes in three cognitive domains: memory, executive function and language (measured by Boston Naming Test). Models were adjusted for age, sex, race, education, *APOE*‐ε4 status, cognitive status, sleep medication use, body mass index, physical activity, depressive symptoms, and cardiovascular risk.

**Result:**

In the fully adjusted models, we found longer night time awakenings were associated with annual declines in memory (β = ‐0.010, 95% CI = ‐0.019 to ‐0.002) and language function (β = ‐0.042, 95% CI = ‐0.072 to ‐0.011). We also found evidence connecting longer sleep duration and greater sleep irregularity with worse memory (β = ‐0.021, 95% CI = ‐0.036 to ‐0.006) and poorer executive function (β = ‐0.019, 95% CI = ‐0.036 to ‐0.003). Both sleep timing (β = ‐0.067, 95% CI = 0.113 to ‐0.022) and sleep irregularity (β = ‐0.138, 95% CI = ‐0.267 to ‐0.009) were associated with annual declines in language function. There was no evidence of effect modification by age, sex, APOE or cognitive status.

**Conclusion:**

Our results suggest that several measures of poor sleep health are associated with 9‐year declines in memory and cognitive performance. Night time awakening length may be a novel indicator of sleep fragmentation worthy of further exploration. Future molecular and mechanistic studies are needed to expand on why sleep fragmentation may contribute to cognitive declines.